# Reversibly locked thionucleobase pairs in DNA to study base flipping enzymes

**DOI:** 10.3762/bjoc.10.239

**Published:** 2014-10-01

**Authors:** Christine Beuck, Elmar Weinhold

**Affiliations:** 1 Department of Structural & Medicinal Biochemistry, University of Duisburg-Essen, Universitätsstr. 2-5, D-45141 Essen, Germany; 2 Institute of Organic Chemistry, RWTH Aachen University, Landoltweg 1, D-52056 Aachen, Germany

**Keywords:** DNA base flipping, DNA cross-link, DNA methyltransferase, nucleic acids, oligodeoxynucleotide, thionucleoside

## Abstract

Covalently interstrand cross-linked DNA is an interesting tool to study DNA binding proteins that locally open up the DNA duplex by flipping single bases out of the DNA helix or melting whole stretches of base pairs to perform their function. The ideal DNA cross-link to study protein–DNA interactions should be specific and easy to synthesize, be stable during protein binding experiments, have a short covalent linker to avoid steric hindrance of protein binding, and should be available as a mimic for both A/T and G/C base pairs to cover all possible binding specificities. Several covalent interstrand cross-links have been described in the literature, but most of them fall short of at least one of the above criteria. We developed an efficient method to site-specifically and reversibly cross-link thionucleoside base pairs in synthetic duplex oligodeoxynucleotides by bisalkylation with 1,2-diiodoethane resulting in an ethylene-bridged base pair. Both linked A/T and G/C base pair analogs can conveniently be prepared which allows studying any base pair-opening enzyme regardless of its sequence specificity. The cross-link is stable in the absence of reducing agents but the linker can be quickly and tracelessly removed by the addition of thiol reagents like dithiothreitol. This property makes the cross-linking reaction fully reversible and allows for a switching of the linked base pair from locked to unlocked during biochemical experiments. Using the DNA methyltransferase from *Thermus aquaticus* (M.TaqI) as example, we demonstrate that the presented cross-linked DNA with an ethylene-linked A/T base pair analog at the target position is a useful tool to determine the base-flipping equilibrium constant of a base-flipping enzyme which lies mostly on the extrahelical side for M.TaqI.

## Introduction

Covalent interstrand DNA cross-links have long sparked clinical and biochemical interest. The cytotoxicity of bisfunctional alkylating agents like nitrogen mustards or chloroethyl nitrosourea (CENU) derivatives has been attributed to their ability to form interstrand DNA cross-links. The resulting covalently linked bases block any machinery that relies on separating the strands of the DNA duplex, e.g., DNA damage repair, replication and transcription [1–5], which is exploited in using CENU derivatives and other interstrand cross-linking reagents as antitumor agents. Chemically synthesized DNA cross-links are of great interest to mimic the reaction products of cross-linking agents acting *in vivo* [6–8] and to mechanistically study enzymes that rely on opening Watson–Crick base pairs by base flipping, as observed with DNA methyltransferases (DNA MTases), DNA glycosylases and some restriction endonucleases, or unwinding the DNA helix with DNA helicases [9–17].

In order to study the binding of these proteins to the cross-linked DNA, the linkage has to be specific and stable under the conditions of the binding experiment, the linker should be short to avoid steric interference with protein binding, and, preferentially, the oligodeoxynucleotides (ODN) should be easily obtained on a DNA synthesizer without the need for synthesizing special building blocks. In fact, a large number of modified building blocks, also suitable for further post-synthetic derivatization, is commercially available and allows a convenient access to modified ODN. Several protocols to generate selective DNA interstrand cross-links in short duplex ODN have been described, however, most of them do not meet all these criteria. Most bisalkylating agents that react with native DNA show selectivity for one type of base pair, e.g., CENU reacts with G/C base pairs, however, regioselective introduction of only one cross-linked base pair at a specified position is not possible. Several linked nucleotides mimicking these alkylation products have been synthesized and then converted into the phosphoamidite building block for chemical DNA synthesis [6–7,18–28]. Not only do these protocols require extensive chemical synthesis, but the sequence of the synthesized duplex ODN cannot be freely chosen since after incorporation of the linked dinucleotide both DNA strands are elongated in parallel and therefore are identical in sequence. If the linked base pair is not incorporated at the 5’ end but in the middle of a duplex, the nucleotides 3' to the linkage in the 2^nd^ strand will need to be filled in with orthogonal 5' to 3' chemistry.

A dT nucleoside analog with an exocyclic aziridine moiety has been chemically incorporated into poly-T ODN. Upon hybridization with a complementary poly-A strand, the aziridine group is attacked by the exocyclic amino group of the opposing adenine, resulting in ring opening and formation of an ethylene cross-linked base pair [29–30]. Other aziridine-substituted nucleobases have been incorporated enzymatically by a DNA polymerase, but elongation past the modified nucleoside has not been reported [31].

Another approach uses nucleobases in which an exocyclic functionality has been substituted by a vinyl group. Upon hybridization with a complimentary, unmodified strand, the vinyl group is attacked by the nucleophilic exo- or endocyclic nitrogen of the partnering base, forming a cross-linked base pair [32–34]. While the cross-linking reaction itself is straight-forward, the vinyl-substituted nucleosides need to be chemically synthesized and subsequently converted into their phosphoamidites for chemical DNA synthesis. The same holds true for cross-links based on aldehyde [8,35–37] or click chemistry [38]. In addition, long linkers that might interfere with protein binding are typically employed.

The Verdine group has developed a cross-linking approach based on the easy postsynthetic introduction of cystamine or longer amino-alkylthiol linkers into ODN using a convertible nucleoside approach [39–48]. The amino-alkylthiol linker is introduced as its disulfide dimer to protect the sulfur and ensure that the reaction with the convertible nucleobase occurs via the amino group. Two of these residues are placed in adjacent base pairs and reduction of the linker disulfides frees the thiol groups, which, upon removal of reducing agent, react under oxidative conditions to form a disulfide cross-link. Adjacent A/A, C/C, G/G and T/T cross-links have been synthesized with this method. An interstrand G/G cross-link of adjacent G/C base pairs was used to study binding of the DNA cytosine-C5 MTase M.HaeIII [39]. The two guanine bases were linked in the minor groove via their exocyclic N2 atoms and the linker does not interfere with M.HaeIII binding. However, for other enzymes with a different specificity or binding mode, especially when attempting to cross-link the target base, the length and steric demand of the linker can be critical and hinder protein binding.

A thionucleobase opposing a guanine base has been cross-linked using a bulky bis-bromoacetamide linker [40–41]. Selective alkylation occurs at the sulfur atom of 4-thiouracil and at the N7 ring nitrogen of the opposite guanine base, introducing a positive charge. This cross-link is not ideally suited to study protein–DNA interactions because the linker is sterically very demanding and the positive charge introduced in the guanine makes it susceptible to depurination.

Direct zero-length cross-linking of two opposing thionucleosides like 2’-deoxy-6-thioinosine (dI^6S^) with 2’-deoxy-4-thiothymidine (dT^4S^) or 2’-deoxy-4-thiouridine (dU^4S^) via a disulfide linkage, appears to be very attractive for studying protein–DNA interactions. These thionucleosides can easily be incorporated in synthetic ODN and the linkage is formed without additional linker atoms avoiding the risk of steric interference with protein binding [42–46]. A disulfide cross-link between 2'-deoxy-6-thioguanosine (dG^6S^) and dU^4S^ was used to study the specificity of the human flap endonuclease FEN1 and confirmed that unpairing the two terminal nucleotides of the DNA duplex is crucial for the selection of the target phosphodiester bond [47]. In our hands though, although direct cross-linking between internal dI^6S^ or dG^6S^ with dU^4S^ residues in duplex ODN could be achieved in yields of up to 80%, the cross-link was not stable during purification. The successful formation of a stable disulfide cross-link might depend on the DNA sequence and position of the thionucleobase pair within the duplex.

We developed a novel method to efficiently cross-link opposite thionucleobases within duplex ODN by exploiting the selective nucleophilic properties of the thiobases. Bisalkylation of a thiobase pair with 1,2-diiodoethane in aqueous solution yields a short linkage with low steric demand. Both A/T and G/C base pair analogs can be site-specifically cross-linked to study enzymes with various sequence specificities. We applied this method to probe the contribution of base flipping to the overall binding affinity of the DNA adenine-N6 MTase from *Thermus aquaticus* (M.TaqI) as an example.

## Results

### Selective cross-linking of thionucleobase pairs in DNA by bis-alkylation with 1,2-diiodoethane

Thionucleobases are excellent soft nucleophiles, and chemoselective alkylation of the sulfur atoms readily occur within the context of duplex DNA without modifying other functionalities [41,48–50]. Reacting a thiobase pair in DNA with a bis-electrophilic linker should thus result in selective cross-link formation. Thionucleosides can easily be incorporated into ODN by using a commercially available cyanoethyl protected phosphoamidite for dG^6S^ and dU^4S^ or by postsynthetic modification of convertible nucleoside precursors for dI^6S^ and dU^4S^ [51].

The ideal geometry of a Watson–Crick base pair within B-DNA is a coplanar orientation of the two nucleobases, stacked between its neighboring base pairs. In contrast, the ideal geometry of saturated linkers will demand non-planar torsion angles. Coleman et al. [44] investigated this topic by performing molecular dynamic simulations for a disulfide cross-linked I^6S^/U^4S^ base pair in B-DNA and showed that the compromise between the dihedrals preferred by the linker and the planar base pair results in local propeller and buckle motions. Since the ethylene linker in our cross-linked base pairs will likely also deviate slightly from a planar arrangement to evade eclipsed conformations, we chose U^4S^ over T^4S^ to avoid a potential steric conflict of the C5 methyl group with the neighboring base pair and keep the local distortion in the DNA helix to a minimum.

**Figure 1:**
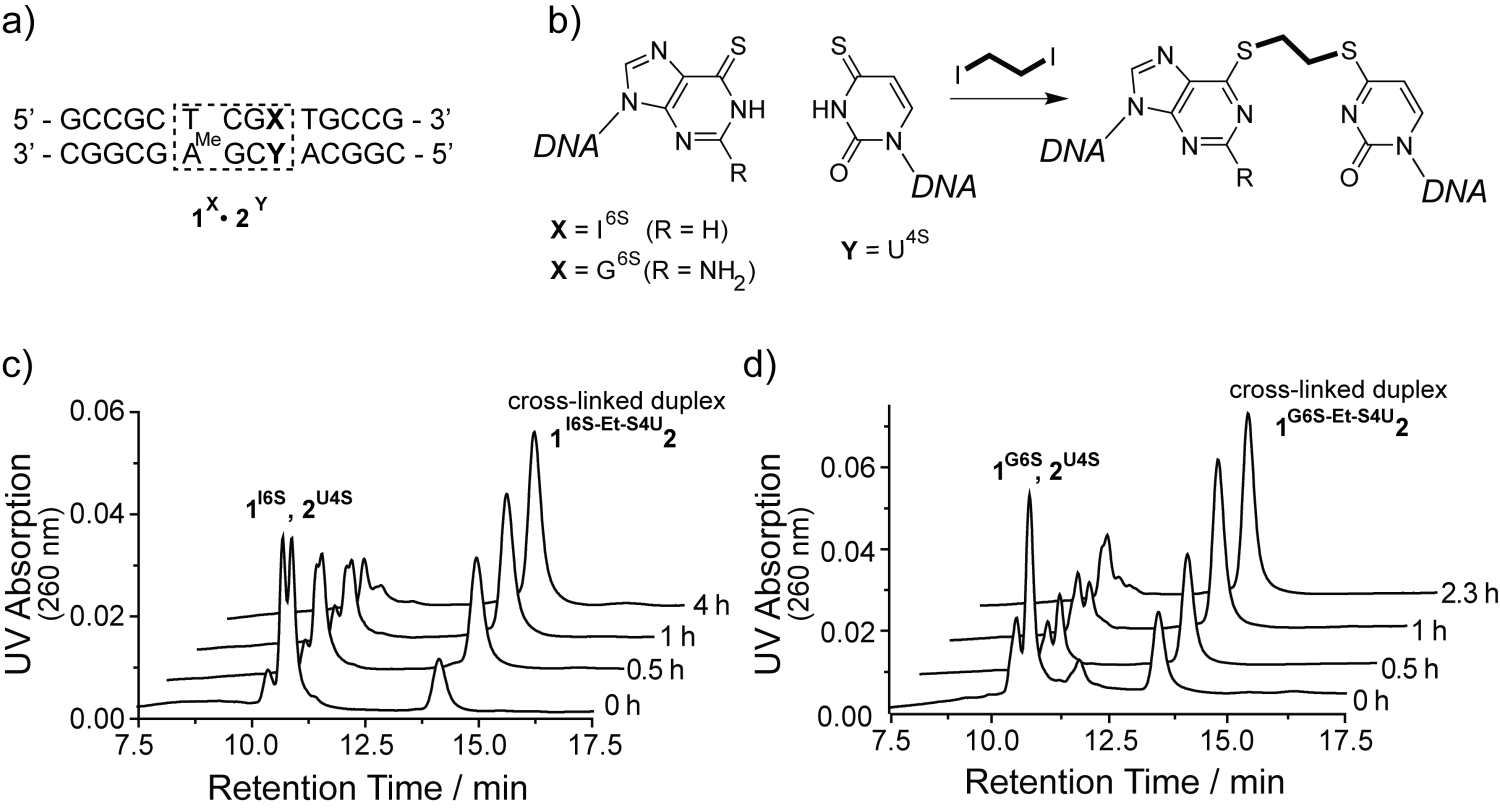
Covalent cross-linking of a I^6S^/U^4S^ or G^6S^/U^4S^ base pair within duplex ODN **1∙2** (a) by bis-alkylation with 1,2-diiodoethane at pH 9.0 (b). The time course for the reaction of duplexes **1^I6S^∙2^U4S^** (c) and **1^G6S^∙2^U4S^** (d) is monitored by denaturing anion exchange HPLC. The non-cross-linked parent duplexes dissociate and elute as their respective single strands, while the covalently linked duplexes elute significantly later.

The 14mer duplex ODN **1^I6S^·2^U4S^** containing a thio-analog of the A/T base pair and **1^G6S^·2^U4S^** containing a thio-analog of the G/C base pair were reacted with 1,2-diiodoethane at room temperature and pH 9.0 (Figure 1a and 1b). The reaction was monitored by denaturing anion exchange chromatography. Addition of urea to the elution buffers and heating of the column to 70 °C causes non-linked duplex ODN to dissociate and elute as their respective single strands while the covalently linked duplexes elute significantly later, comparable to a single-stranded ODN of twice the length (Figure 1c and 1d). In contrast, no species eluting at higher retention times are observed when each of the single strands alone is reacted with 1,2-diiodoethane. These observations are consistent with the formation of the interstrand cross-linked duplexes **1^I6S-Et-S4U^2** (80% yield after 4 h) and **1^G6S-Et-S4U^2** (72% after 2.3 h). The cross-linked duplexes exhibit a significantly diminished UV absorption at 332 nm compared to the duplex ODN with unmodified thionucleobases. The long wavelength absorption band of thionucleobases is characteristic for their thio–keto tautomer [52]. This band is shifted towards shorter wavelengths upon alkylation at the sulfur atom and loss of the carbon–sulfur double bond. A small fraction of single-stranded ODN with low 332 nm absorption remains and can be assigned to alkylation products where two 1,2-diiodoethane molecules reacted with both thionucleobases in the duplex or where the reaction with 1,2-diiodoethane took place on only one of the thionucleobases but failed to react with the opposing base.

**Figure 2:**
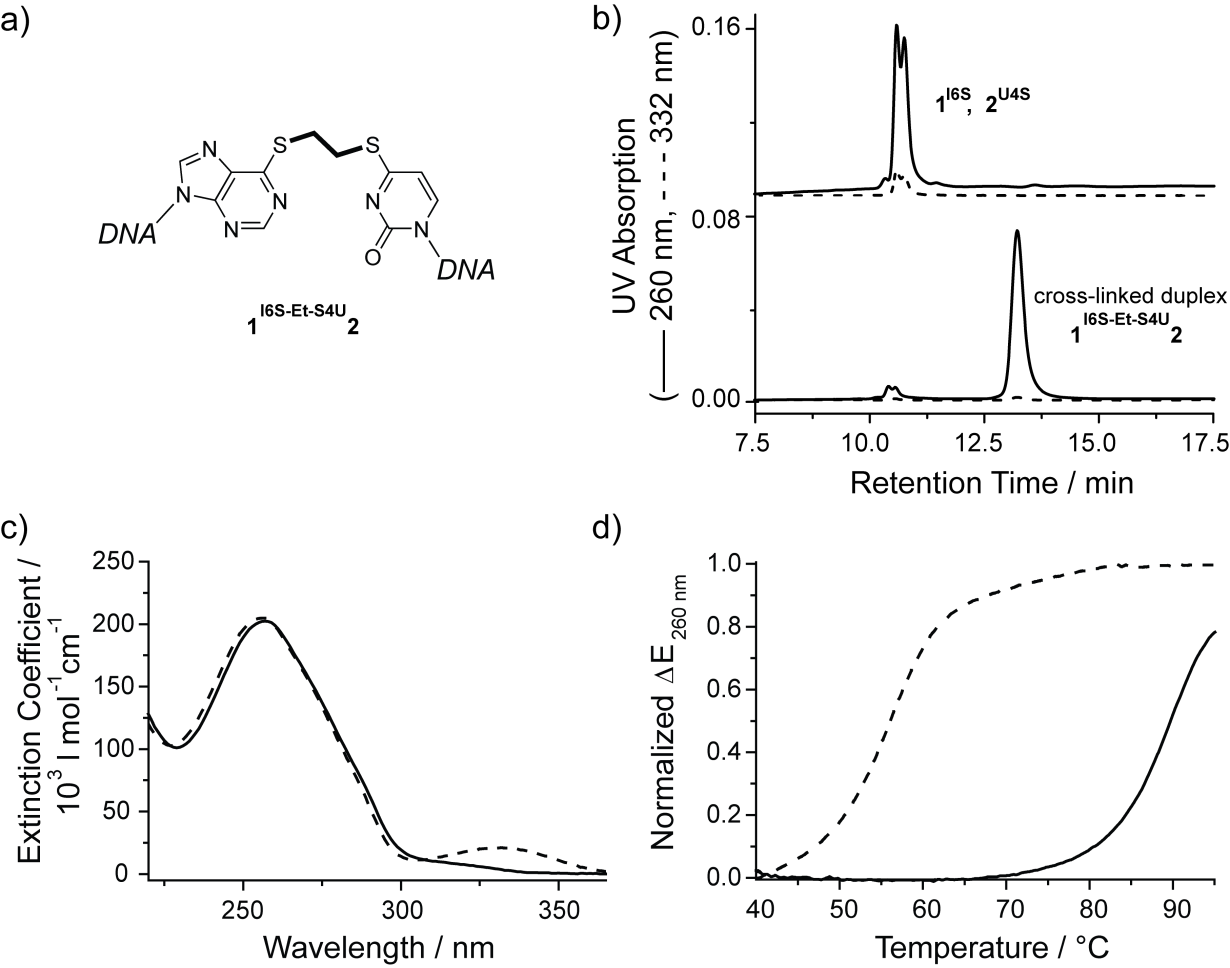
Characterization of HPLC-purified cross-linked duplex **1^I6S-Et-S4U^2** (a) by denaturing anion exchange HPLC (b, bottom) in comparison to the non-linked parent duplex (b, top). Alkylation at the sulfur atoms within the thio-nucleobases is confirmed by the wavelength shift and strong intensity reduction of the absorption band above 300 nm in the UV spectrum (c, solid) compared to the spectrum of **1^I6S^∙2^U4S^** (dashed). The thermal stability of the cross-linked duplex **1^I6S-Et-S4U^2** (d, solid) is drastically increased compared to **1^I6S^∙2^U4S^** (dashed).

**Figure 3:**
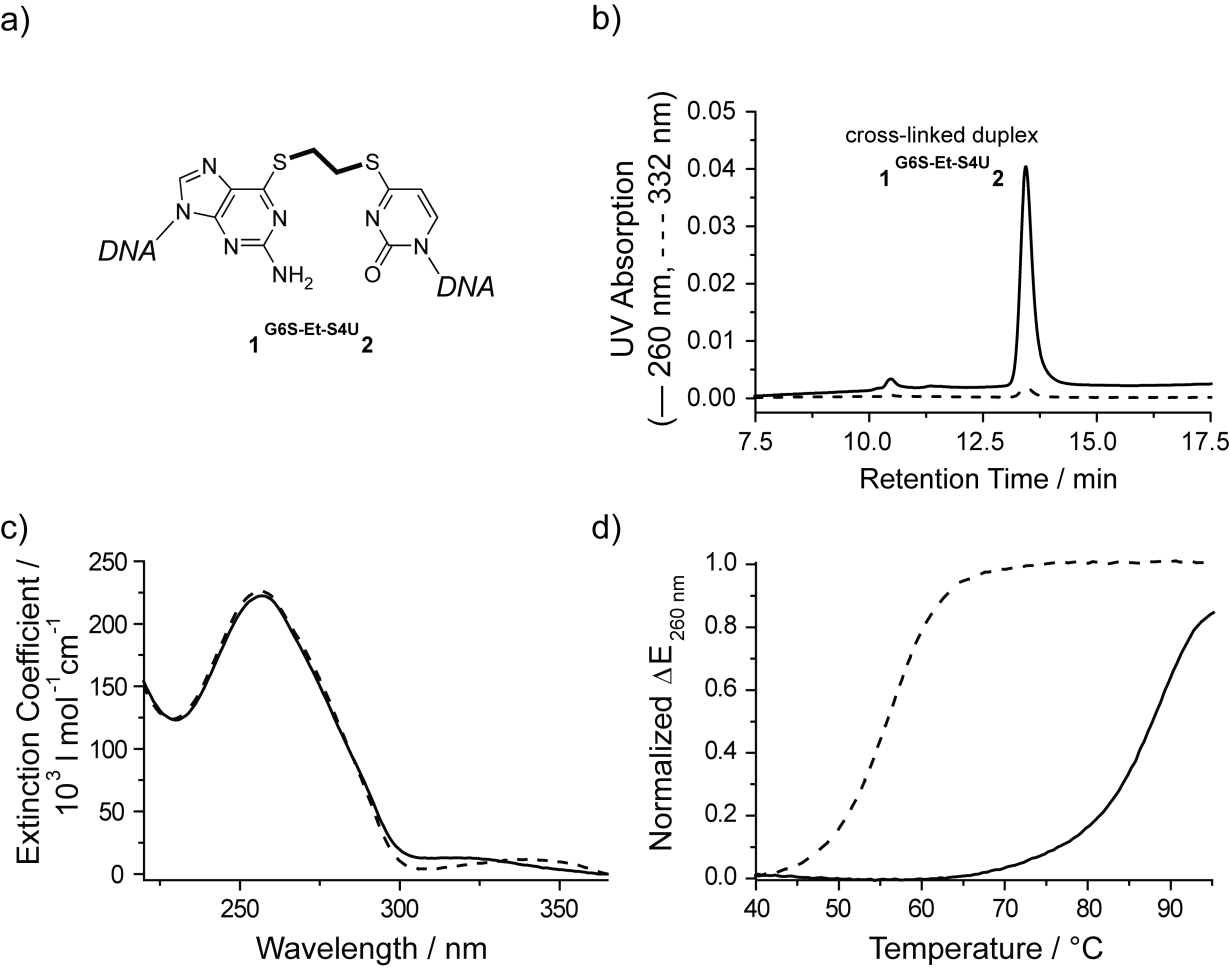
Characterization of HPLC-purified cross-linked duplex **1^G6S-Et-S4U^2** (a) by denaturing anion exchange HPLC (b). Alkylation at the sulfur atoms within the thionucleobases is confirmed by the strong reduction of the absorption band above 300 nm in the UV spectrum of **1^G6S-Et-S4U^2** (c, solid) compared to the spectrum of **1^G6S^∙2^U4S^** (dashed). The thermal stability of the cross-linked duplex **1^G6S-Et-S4U^2** (d, solid) is drastically increased compared to **1^G6S^∙2^U4S^** (dashed).

Both cross-linked duplexes **1^I6S-Et-S4U^2** and **1^G6S-Et-S4U^2** were isolated by preparative denaturing anion exchange HPLC. An aliquot of each cross-linked duplex was resuspended in buffer without reducing agent. The integrity of the purified cross-linked duplexes was assessed by denaturing HPLC (Figure 2b and Figure 3b). Both cross-linked duplexes elute at 13.4 min and show the characteristic low 332 nm absorption, with only trace amounts of dissociated single strands in the 10.5–10.7 min range. The elution profile did not change over the course of 3 days showing that the cross-link is stable at room temperature in the absence of reducing agents. The UV spectrum of both cross-linked duplexes **1^I6S-Et-S4U^2** and **1^G6S-Et-S4U^2** clearly confirms the shift of the thio-nucleobase absorption band over 300 nm towards lower wavelengths as it is typical for S-alkylated thio-nucleobases (Figure 2c and Figure 3c). Circular dichroism [53] spectroscopy is sensitive to the overall global conformation of nucleic acids. The CD spectrum of duplex ODN **1^I6S-Et-S4U^2** retains the characteristic shape of B-DNA, showing that the ethyl cross-link does not significantly distort the DNA helix (data not shown). The cross-linked duplexes were further characterized by thermal denaturation monitoring the UV absorption at 260 nm (Figure 2d and Figure 3d). A covalent link between the two strands within a DNA duplex is expected to locally stabilize the DNA duplex by preventing the complete dissociation of its strands. Ultimately, at high temperatures all hydrogen-bonded base pairs will be disrupted even in the presence of a covalent link. Therefore, a shift of the melting transition towards higher temperatures is expected for the cross-linked duplexes. The non-cross-linked duplex ODN **1^I6S^·2^U4S^** and **1^G6S^·2^U4S^** cooperatively melt at 56 °C, which is comparable to the melting temperature of the same duplex with a natural mismatched base pair, while the 14mer duplex ODN with a native hydrogen-bonded A/T base pair melts at 66 °C. The thionucleobases behave like a mismatch because both thionucleobases preferably exist in their thio-keto form and thus no Watson–Crick hydrogen bonds are formed in the I^6S^/U^4S^ pair and only one is possible in the G^6S^/U^4S^ pair. Both cross-linked duplexes have melting temperatures of 88–89 °C demonstrating a drastic stabilization compared to the parent duplex ODN with the unmodified thionucleobases and the matched duplex ODN. This 33 °C increase in the melting temperature upon cross-linking is comparable to the stabilization previously observed for other interstrand cross-links [44,54].

We have shown that duplex ODN containing a thionucleobase pair can easily be covalently cross-linked by bis-alkylation with 1,2-diiodoethane. Both duplexes with a linked A/T and G/C base pair analog can be obtained by choosing the corresponding I^6S^/U^4S^ or G^6S^/U^4S^ thionucleobase pair. The cross-linked duplexes can be isolated and were stable in the absence of reducing agent.

### Traceless linker removal with thiol nucleophiles

**Figure 4:**
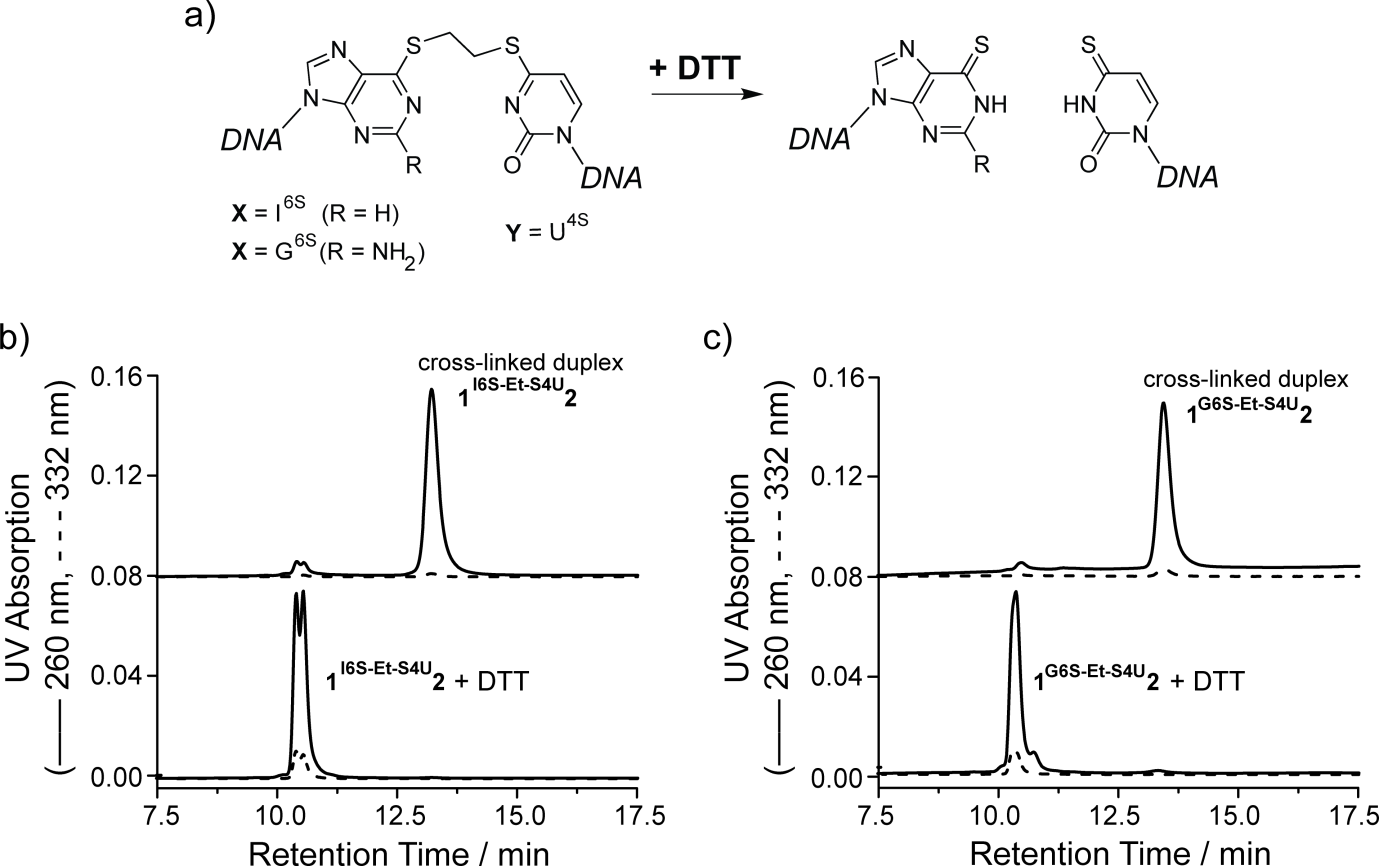
Opening and traceless linker removal of the cross-linked duplexes **1^I6S-Et-S4U^2** and **1^G6S-Et-S4U^2** by adding DTT (a). Denaturing anion exchange HPLC chromatograms (b, c) demonstrate that the reaction products dissociate and elute at the same retention times as their parent single strands. Furthermore, the UV absorption at 332 nm is fully recovered, indicating that the thio-keto function in the nucleobases has been restored.

The covalent ethylene cross-links in **1^I6S-Et-S4U^2** and **1^G6S-Et-S4U^2** are stable at room temperature in the absence of thiol reagents. Interestingly, when an aliquot of each cross-linked duplex is resuspended in buffer with 1 mM dithiothreitol (DTT) and analyzed by denaturing anion exchange HPLC, no more cross-linked duplex (retention time 13.4 min) is observed. Instead, the duplex dissociates and the single strands elute at retention times between 10.5–10.7 min. These single strands exhibit a fully restored absorption at 332 nm, suggesting that the cross-linking reaction was reversed and the duplex ODN **1^I6S^∙2^U4S^** and **1^G6S^∙2^U4S^** with the thionucleosides in their thio-keto form have been restored (Figure 4). The UV spectra of the cross-linked duplex **1^I6S-Et-S4U^2** before and after addition of 1 mM DTT show the full reappearance of the band at long wavelength around 330 nm identical to the unlinked duplex **1^I6S^∙2^U4S^**, corroborating that the linker was fully removed from both thionucleobases.

In order to demonstrate that the opened duplex in fact contains the unmodified thionucleosides, the duplex ODN resulting from the reaction of **1^I6S-Et-S4U^2** with DTT was transferred into DTT-free buffer using a desalting column and the cross-linking reagent 1,2-diiodoethane was added again. After 2.5 h the reaction was analyzed by denaturing anion exchange chromatography, showing the reappearance of the cross-linked duplex (data not shown).

**Figure 5:**
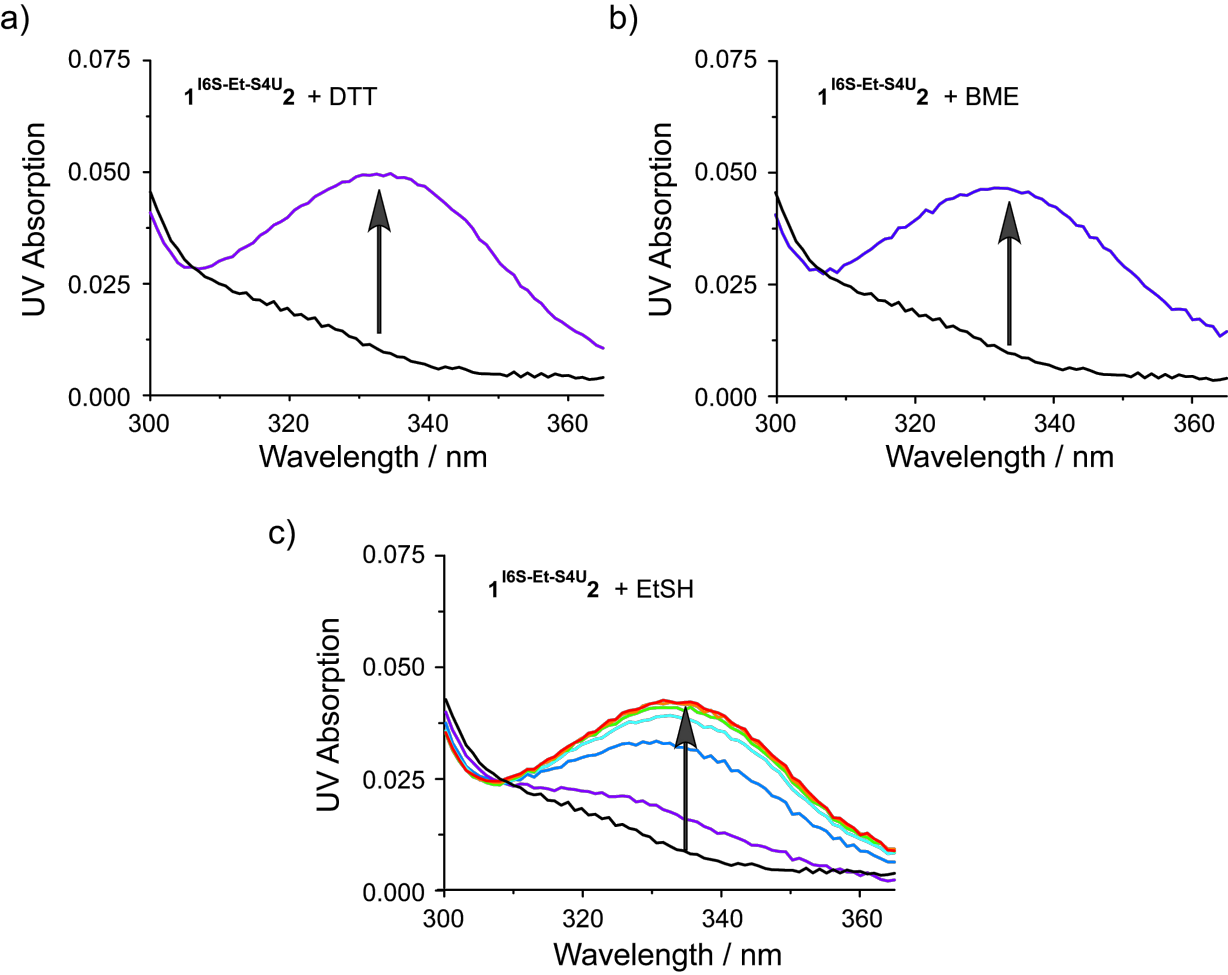
UV time course of opening of the cross-link in duplex **1^I6S-Et-S4U^2** with the thiol nucleophiles DTT (a), β-mercaptoethanol (BME, b) and ethanethiol (EtSH) (c). Upon removal of the linker, the thionucleobase absorption bands above 300 nm are restored. Black, no nucleophile; purple, 0 min; blue, 5 min; cyan, 10 min; green, 15 min; orange, 20 min; red, 25 min.

Opening of the cross-linked base pair was further examined with other thiol nucleophiles like β-mercaptoethanol (BME) and ethanethiol (EtSH) in addition to DTT. These thiols were added to a solution of the cross-linked duplex **1^I6S-Et-S4U^2** and the reaction was monitored by recording the UV spectrum as a function of time (Figure 5). DTT and BME are able to remove the alkyl linker and restore the thionucleobase specific UV absorption above 300 nm almost instantaneously (Figure 5a and 5b) while the reaction with EtSH proceeds slower with a half life of less than 5 min (Figure 5c).

We conclude that reversion of the cross-linking reaction occurs by attack of a thiol nucleophile onto the carbon atoms of the linker with the thionucleobases acting as the leaving groups. The re-opened reaction product can be re-cross-linked by renewed addition of 1,2-diiodoethane after removing any thiol reagent (not shown).

While the duplex ODN with an ethylene-linked thionucleobase pair are stable in the absence of reducing agents, the addition of thiol-containing nucleophiles such as DTT quickly and completely removes the ethylene bridge from both thionucleobase pairs. This interesting feature of quick traceless linker removal allows for a switching of the thionucleobase pair from the locked to the unlocked state.

### Cross-linked DNA as a tool to determine the base flipping equilibrium of DNA-modifying enzymes

**Figure 6:**
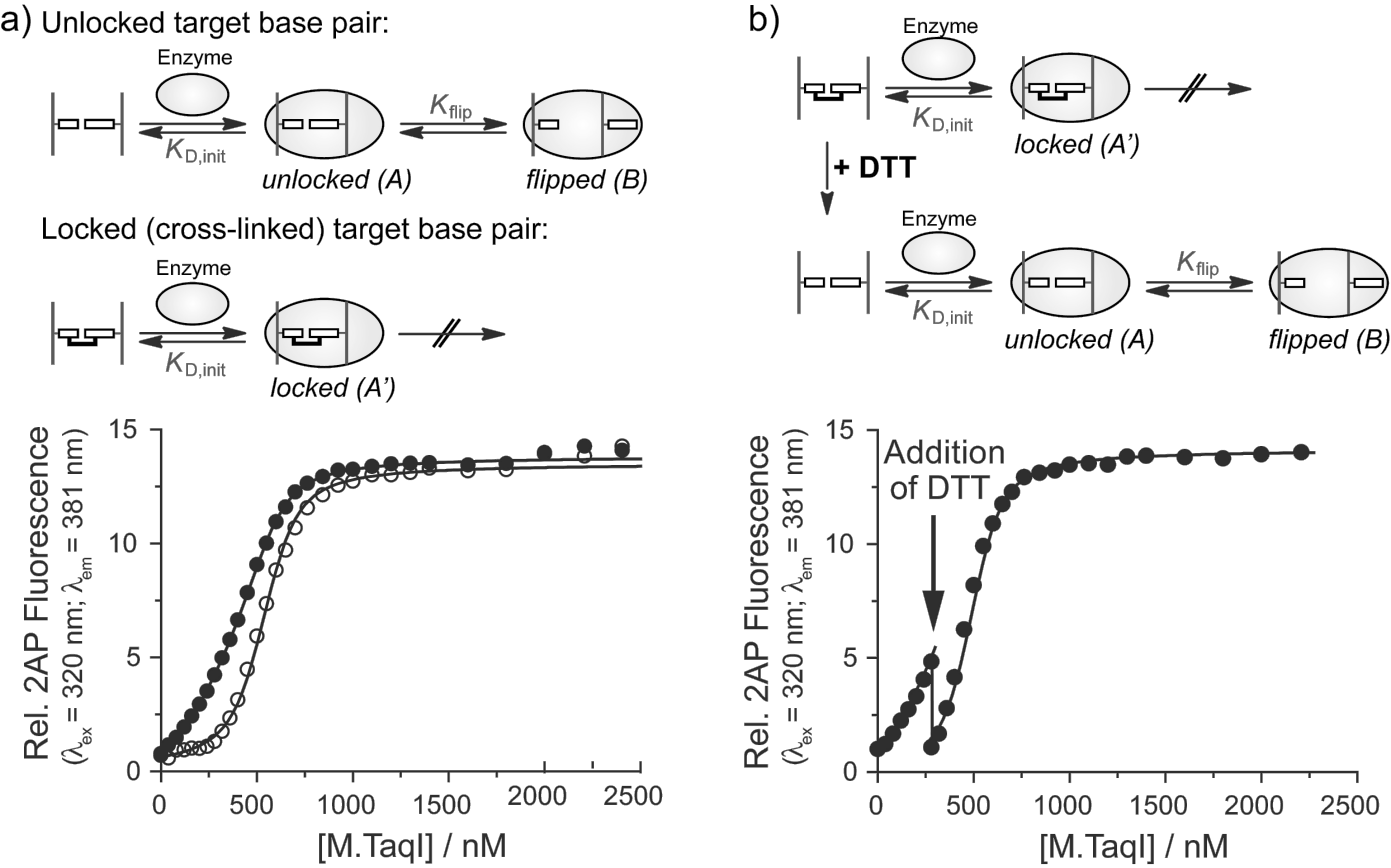
Competition binding of M.TaqI to DNA with unlocked and locked target base pairs. Increasing amounts of M.TaqI were added to a mixture of the DNA of interest and a fluorescent DNA with known *K*_D_ that contains the target base analog 2-aminopurine (2AP). A more delayed increase in the 2AP fluorescence indicates tighter binding of the non-fluorescent DNA. (a) Binding of M.TaqI to duplex **1^I6S^∙2^U4S^** with unlocked target base pair (top reaction scheme and open circles) allows base flipping of the target base into the active site. The observed dissociation constant 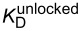 comprises both the initial binding constant *K*_D,init_ and the base flipping equilibrium constant *K*_flip_. Binding of M.TaqI to the cross-linked duplex **1^I6S-Et-S4U^2** with locked target base pair (bottom reaction scheme and closed circles) prevents base flipping, and the observed dissociation constant 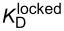 equals *K*_D,init_. (b) Switching from locked to unlocked target base pair during the M.TaqI binding experiment. The same competition titration was started with the cross-linked duplex **1^I6S-Et-S4U^2** in buffer without reducing agent preventing base flipping. Upon addition of DTT (arrow) the cross-link is unlocked enabling base flipping and thus tighter binding. The titration is continued and now follows the binding curve for the duplex with unlocked target base pair.

DNA-modifying enzymes often use a base flipping mechanism to gain access to their target bases [9,11–17,55–68]. Experimental evidence supports a two-step binding model comprised of an initial association equilibrium with the dissociation constant *K*_D,init_, followed by flipping of the target base with the equilibrium constant *K*_flip_ (Figure 6a) [55–56,69]. The binding affinity of base-flipping enzymes is often determined using DNA with the fluorescent base analog 2-aminopurine (2AP) at the target site [70–76]. The fluorescence of 2AP is quenched within the base stack of duplex DNA and increases once the DNA is bound and the 2AP base is flipped out of the DNA helix by the base-flipping enzyme. A competition binding assay is used to determine the dissociation constant *K*_D_ for a non-fluorescent DNA substrate. Titrating enzyme into a fixed ratio of the fluorescent DNA with known *K*_D_ and the non-fluorescent DNA leads to a fluorescence intensity increase which in turn depends on the concentrations and ratio of binding affinities. The contribution of the base flipping step (*K*_flip_) to the overall observed binding affinity cannot be extracted from these data because the non-flipped and flipped enzyme complexes A and B (Figure 6) cannot be distinguished in a single binding experiment. Therefore the overall dissociation constant 
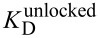
 is given by (see Supporting Information File 1):

[1]
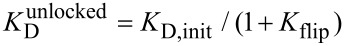


In order to calculate *K*_flip_, the dissociation constant *K*_D,init_ of the initial encounter complex A must be determined independently. Utilizing DNA with a covalently locked target base pair (Figure 6) the base flipping step is blocked and the dissociation constant 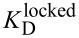 is equal to the dissociation constant of the initial complex A’:

[2]
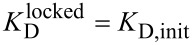


It is of great importance that the presence of the linker does not impair initial binding so that *K*_D,init_ is identical for the formation of both complexes A and A’. When both binding experiments with locked and unlocked target base pair are performed, *K*_flip_ is obtained by combination of equations (1) and (2):

[3]
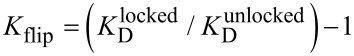


We used the duplex **1^I6S-Et-S4U^2** containing a locked A/T base pair analog at the target position within the double-stranded 5’-TCGA-3’ recognition site of the DNA adenine-N6 MTase M.TaqI to determine the equilibrium constant *K*_flip_ for the base flipping step. The dissociation constants for the duplexes with an unlocked (**1^I6S^·2^U4S^**) and with a locked (**1^I6S-Et-S4U^2**) target base pair analog were determined by a competitive 2AP fluorescence binding assay in separate experiments (Figure 6a). With 
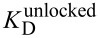
 = 1.0 ± 0.5 nM and 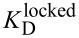 = 6.0 ± 3 nM equation (3) provides *K*_flip_ = 5, corresponding to about 80% of the target base being flipped within the M.TaqI-DNA complex. Therefore, the base flipping equilibrium of M.TaqI strongly lies on the extrahelical side.

To demonstrate that the locked target base pair can be re-opened during the binding experiment, the M.TaqI titration was started with cross-linked duplex ODN **1^I6S-Et-S4U^2** in buffer without reducing agent and M.TaqI was added up to the point where the binding curves for the duplexes with unlocked and locked target base pair (Figure 6a) differed the most. DTT was then added to unlock the target base pair, allowing base flipping to take place (Figure 6b). The binding affinity is thus improved resulting in more potent competition and a significant drop in 2-AP fluorescence intensity. Continuing the titration with increasing M.TaqI concentrations, the binding curve now follows the one observed with duplex **1^I6S^·2^U4S^** with an unlocked target base pair.

Cross-linked duplex DNA with an ethylene-linked thio-base pair has proven to be a useful tool for analyzing the base flipping equilibrium of the DNA MTase M.TaqI. The target base within the M.TaqI-DNA complex is mostly in the extrahelical conformation which places it into the enzyme's active site. The quick and traceless removal of the linker by addition of DTT enables switching of the target base pair from a locked to an unlocked state during the course of the binding experiment.

## Discussion

Here we presented a new and convenient method to engineer a reversibly locked thionucleobase pair in duplex DNA by bisalkylation of either an I^6S^/U^4S^ or G^6S^/U^4S^ pair with 1,2-diiodoethane. There are several advantages of the presented cross-linking protocol over most others known in the literature: The cross-link is introduced postsynthetically on the level of duplex DNA in aqueous solution, without the need of extensive chemical synthesis and with no restrictions with respect to the DNA sequence. The cross-linked analogs of both an A/T and a G/C base pair can be obtained by starting with either an I^6S^/U^4S^ or G^6S^/U^4S^ base pair, and therefore, this chemistry can be employed to study any DNA-modifying enzyme no matter what target base specificity it possesses. While different linker geometries might work for different DNA-binding proteins, the ethyene-linked thiobase pairs offer the advantage of a very short linker with little steric demand for studying DNA-opening enzymes. The cross-linked duplex ODN are stable at room temperature in buffers without thiol reagents. Addition of thiol nucleophiles results in a quick and traceless removal of the ethylene linker which makes them interesting tools for chemical switching DNA from locked to unlocked base pairs during biophysical experiments. However, this interesting property requires that all proteins studied are stable in buffers without reducing thiol reagent. Even small amounts of these nucleophiles from the protein stock solutions are sufficient to open the linkage.

As a proof of principle, we applied a duplex ODN with an ethyl cross-linked I^6S^/U^4S^ base pair as A/T pair analog within the 5’-TCGA-3’ recognition sequence of the DNA MTase M.TaqI and determined the base flipping equilibrium constant *K*_flip_ within the M.TaqI-DNA complex. DNA with a locked target base pair allows initial binding of the enzyme to the DNA but prevents subsequent flipping of the target base. *K*_flip_ can therefore be calculated from two binding experiments, one with an unlocked I^6S^/U^4S^ pair, giving the overall binding constant that comprises both binding and base flipping, and one with the locked cross-linked target base pair (Figure 6), which reports the initial binding only. The resulting *K*_flip_ = 5 demonstrates that about 80% of the M.TaqI target base are found in the extrahelical conformation within the M.TaqI-DNA complex.

Since there are some chemical differences between an I^6S^/U^4S^ pair and the native A/T target base pair the value obtained for *K*_flip_ should be interpreted as an approximation for the base flipping equilibrium with the native DNA target. In the I^6S^/U^4S^ base pair, both thiobases exist in their thio-keto form and therefore cannot form hydrogen bonds like the natural A/T base pair. Without having to expend the energy to break any hydrogen bonds, flipping the target base should be easier, but at the same time, the I^6S^ base is not able to form the hydrogen bonds in the active site of the enzyme that are observed in the complex with the native adenine target [77]. Comparing binding of M.TaqI to its native hemi-methylated target DNA and the fully methylated DNA (Figure S1, Supporting Information File 1), it is expected that the methylated target base, which is the product of the DNA MTase reaction, sterically interferes with residues in the active site of the enzyme, resulting in favoring the innerhelical target base conformation which helps to release the reaction product from the enzyme. Under the assumption that the base flipping equilibrium lies completely on the innerhelical side for the fully methylated reaction product and using the dissociation constants for hemi-methylated DNA (*K*_D_ = 2.9 ± 1.5 nM) and fully methylated DNA (*K*_D_ = 56 ± 30 nM), one obtains *K*_flip_ = 18 for the hemi-methylated DNA-M.TaqI complex. This corresponds to 95% of the target adenine being in the extrahelical conformation which is similar to the 80% extrahelical target base obtained with the A/T base pair analog. Interestingly, the fully methylated duplex binds even worse than the cross-linked duplex (Figure S1, Supporting Information File 1) which suggests that the methyl group in the natural reaction product *N*6-methyladenine (A^Me^) does not only destabilize the extrahelical conformation in the flipped but also in the non-flipped (initial) complex. This may further enhance product release and reduce product inhibition. The cross-linked target base pair is missing the methyl group (Figure S2, Supporting Information File 1) and might therefore be a better mimic for the substrate with innerhelical target base than for the methylated reaction product.

Many other proteins locally open up the DNA duplex to perform their function, ranging from flipping a single nucleobase out of the DNA helix to melting whole stretches of base pairs. A base flipping mechanism is utilized by many enzymes that either chemically modify the flipped nucleobase or repair a lesion, e.g., other DNA methyltransferases [9–10], DNA demethylases like the AlkB family [11], DNA alkyltransferases that are essential to DNA alkylation damage repair [12–13], DNA glycosylases from the base excision repair pathway [14–16], and photolyases repairing UV damage in DNA [17]. DNA helicases, which locally separate the two DNA strands, are important to enable vital cellular processes like DNA replication, DNA repair, chromatin remodeling and telomere maintenance [78–81]. Cross-linked DNA will not only provide a useful tool to study DNA binding and base flipping thermodynamics, as we demonstrated for the DNA MTase M.TaqI, but could also be used to determine their site and mechanism of action by introducing cross-linked base pairs at different positions within the DNA, or stall proteins on the DNA in a pre-flipped complex for structure determination to reveal the initial contacts between the proteins and their DNA targets that are formed before the target base is flipped or strands are separated.

## Conclusion

In conclusion, we have developed a new efficient method to site-specifically and reversibly cross-link thionucleoside base pairs in duplex DNA via an ethylene bridge. Both linked A/T and G/C base pair analogs can easily be prepared which allows studying any base flipping enzyme regardless of its sequence specificity. We demonstrated that cross-linked DNA is a useful tool to study base flipping enzymes and proved that the base flipping equilibrium lies mostly on the extrahelical side and thus has an important contribution to the overall DNA-binding energy of M.TaqI.

## Experimental

Solid-phase DNA synthesis was performed on an ABI DNA Synthesizer 392 in 1 µmol scale following the standard cycles recommended by Applied Biosystems with a coupling time of 30 s for the natural nucleotides. For unnatural nucleotides, the coupling time was extended to 90 s. Fast deprotectable *tert*-butylphenoxyacetyl (TAC) protected A, C and G phosphoamidites and coupling reagents including TAC anhydride as capping reagent were purchased from Proligo. *N*6-Methyl-2’-deoxyadenosine (A^Me^), 6-thio-2’-deoxyguanosine (G^6S^) phosphoamidites, as well as the convertible thionucleoside precursor phosphoamidites *O*6-phenyl-2'-deoxyinosine (I^OPh^) and 4-triazolyl-2'-deoxyuridine (U^Tri^) were purchased from Glen Research. Oligodeoxynucleotides (ODN) with G^6S^ and A^Me^ were deprotected according to the manufacturer's protocol. ODN with convertible nucleosides I^OPh^ and U^Tri^ were deprotected and converted to their thionucleoside containing counterparts as described in [51]. Duplexes were annealed before use at a concentration of 100 µM in the respective experiment buffer by heating to 95 °C for 2 min followed by slow cooling to room temperature.

**Table 1:** ODN sequences used in this study. The M.TaqI recognition sequence is highlighted in bold. A^Me^ = *N*6-methyladenine.

ODN	Sequence

**1^I6S^**	5'- GCCGC **TCGI^6S^** TGCCG -3'
**1^G6S^**	5'- GCCGC **TCGG^6S^** TGCCG -3'
**1^A^**	5'- GCCGC **TCGA** TGCCG -3'
**1^AMe^**	5'- GCCGC **TCGA^Me^** TGCCG -3'
**2^U4S^**	5'- CGGCA **U^4S^CGA^Me^** GCGGC -3'
**2^T^**	5'- CGGCA **TCGA^Me^** GCGGC -3'

The DNA MTase M.TaqI was overexpressed and purified as described before [57,77]. For binding experiments with cross-linked duplex ODN, M.TaqI was transferred into storage buffer without reducing agent.

### Denaturing anion exchange HPLC

Denaturing anion exchange HPLC was performed on a Perseptive Poros HQ 10 column (10 × 100 mm, 10 µm) at a flow rate of 2 mL/min. Buffer A consist of 10 mM Tris/HCl, pH 7 and 5 M urea. Buffer B consists of 10 mM Tris/HCl, pH 7, 1 M potassium chloride and 5 M urea. The column was heated in a water bath to 70 °C. Before entering the column, the buffer was pre-heated by passing it through a 0.5 m steel capillary placed in the 70 °C water bath. The DNA was eluted with 20% B (0–5 min), followed by linear gradients with 20–50% B (5–10 min), and 50–65% B (10–20 min). UV absorption was detected at 254 nm and 332 nm. The non-cross-linked parent duplexes dissociate and elute as their respective single strands, while the covalently linked duplexes elute significantly later. HPLC-purified cross-linked duplexes were desalted using a NAP-5 gel filtration column as described below.

### UV spectroscopy

UV spectroscopy was performed using a Varian CARY 3E UV–Vis spectrometer in a 1 cm quartz cuvette. The concentration of ODN was determined in water at 260 nm and 25 °C using the nearest neighbor method [82] to calculate the extinction coefficients. *N*6-Methyl-2’-deoxyadenosine was treated as 2’-deoxyadenosine. For dG^6S^, an extinction coefficient of 8,000 L mol^−1^ cm^−1^ at 260 nm was derived from an experimental spectrum of the free nucleoside and the published extinction coefficient of 24,800 L mol^−1^ cm^−1^ at 342 nm [83]. For dI^6S^ and dU^4S^ the low absorption at 260 nm was neglected.

UV spectra of duplex ODN were recorded from 220 nm to 365 nm at a DNA concentration of 5 µM in phosphate buffer (10 mM NaPi, pH 7.0, 100 mM NaOAc).

Melting curves of duplex ODN (2.5 µM) in phosphate buffer (10 mM NaPi, pH 7.0, 100 mM NaOAc) were recorded at 260 nm between 40 °C and 95 °C in 0.1 K steps at a heating rate of 0.5 K/min. Melting temperatures *T*_M_ were obtained as the maximum of the first derivative of the melting curve. Since the melting curves for the cross-linked DNA did not reach their plateau, amplitudes were normalized setting the lowest data point to 0 and the inflection point [84] to 0.5.

### Time course of cross-linking reactions with diiodoethane

Duplex ODN **1^I6S^·2^U4S^** or **1^G6S^·2^U4S^** (77 µM) were annealed in reaction buffer (50 µL; 20 mM Tris/HOAc pH 9.0, 10 mM Mg(OAc)_2_, 50 mM KOAc) and a solution of 1,2-diiodoethane (20 µL, 33 mM) in DMF was added. Slight precipitate of 1,2-diiodoethane re-dissolved upon further mixing. The reaction mixture was incubated at room temperature in the dark. Samples (10 µL) were analyzed after 0 h, 0.5 h, 1 h and 2.3 h or 4 h reaction time by denaturing anion exchange HPLC. The non-cross-linked parent duplexes **1^I6S^·2^U4S^** or **1^G6S^·2^U4S^** dissociate into their respective single strands **1^I6S^** or **1^G6S^** and **2^U4S^** eluting at 10.5–10.7 min. Cross-linked duplexes **1^I6S-Et-S4U^2** (*t*_R_ = 13.4 min) and **1^G6S-Et-S4U^2** (*t*_R_ = 13.4 min) elute at significantly higher retention times. After 4 h or 2.3 h, no non-alkylated single strands could be observed, and cross-linked duplexes **1^I6S-Et-S4U^2** and **1^G6S-Et-S4U^2** were formed with 80% or 72% yield, respectively. Remaining amounts of ODN eluting around 10.5 min showed low 332 nm absorption and were attributed to S-alkylated single strands.

#### Preparative cross-linking experiments

Duplex ODN **1^I6S^·2^U4S^** or **1^G6S^·2^U4S^** (77 µM) were annealed in reaction buffer (100 µL; 20 mM Tris/HOAc pH 9.0, 10 mM Mg(OAc)_2_, 50 mM KOAc) and a solution of 1,2-diiodoethane (40 µL, 33 mM) in DMF was added. Slight precipitate of diiodoethane re-dissolved upon further mixing. The reaction mixture was incubated at room temperature in the dark. After 4 h, the cross-linked duplexes **1^I6S-Et-S4U^2** (*t*_R_ = 13.4 min) and **1^G6S-Et-S4U^2** (*t*_R_ = 13.4 min) were isolated by denaturing anion exchange HPLC. Purified cross-linked duplexes were desalted using illustra NAP-5 gel filtration columns (GE Healthcare). The columns were drained from storage solution by gravity flow and equilibrated with H_2_O (10 mL). Combined HPLC fractions (0.5 mL) were applied to the column and allowed to enter the gel bed. The DNA was eluted with H_2_O (1 mL), and the concentration of the eluate was determined by UV spectroscopy. The integrity of the cross-linked duplexes was confirmed by analytical denaturing anion exchange HPLC. The desalted duplexes were aliquoted (1 nmol), immediately lyophilized to dryness and stored at −80 °C.

#### Linker removal from cross-linked duplex **1^I6S-Et-S4U^2** with different thiol nucleophiles

Lyophilized **1^I6S-Et-S4U^2** (1 nmol) was resuspended in buffer (400 µL; 20 mM Tris/HOAc pH 9.0, 10 mM Mg(OAc)_2_, 50 mM KOAc). Dithiothreitol (DTT, 1 mM), β-mercaptoethanol (BME, 2 mM), ethanethiol (EtSH, 2 mM), or no thiol reagent were added. UV spectra were recorded (DTT: 0 min; BME: 0 min, EtSH: 0–25 min in 5 min increments) from 300 nm to 365 nm to monitor the reappearance of the band >300 nm which is characteristic for the non-alkylated thionucleobases. At the end of each time course, the reaction mixtures (200 µL each) were analyzed by denaturing anion exchange HPLC to confirm the opening of the cross-link.

#### Determination of dissociation constants for M.TaqI-DNA complexes

Binding affinities of the non-fluorescent duplex **1^I6S^·2^U4S^** with an unlocked and the cross-linked duplex **1^I6S-Et-S4U^2** with a locked target base pair, as well as the hemimethylated native substrate **1^A^·2^T^** and the fully methylated duplex **1^AMe^·2^T^** were determined in a competitive fluorescence binding assay using a 36mer duplex ODN containing the fluorescent base analog 2-aminopurine (2AP) at the target position within the recognition sequence of M.TaqI [73]. Fluorescence titrations were performed with a Varian Cary Eclipse fluorescence spectrophotometer using a 10 × 10 mm quartz cuvette at 25 °C with an excitation wavelength of 320 nm and an emission wavelength of 381 nm.

Binding of M.TaqI to duplexes **1^I6S^·2^U4S^** and **1^I6S-Et-S4U^2**: To a solution (600 µL) of 36mer duplex with 2AP at the target position (200 nM) and duplex **1^I6S^·2^U4S^** or **1^I6S-Et-S4U^2** (400 nM) in M.TaqI binding buffer (20 mM TrisOAc, 10 mM Mg(OAc)_2_, 50 mM KOAc, pH 7.9, 0.01% reduced Triton X-100) and either 1 mM DTT (**1^I6S^·2^U4S^**) or no reducing agent (**1^I6S-Et-S4U^2**) were added stepwise increasing amounts of a solution containing M.TaqI (10 µM), 36mer duplex with 2AP (200 nM) and a duplex **1^I6S^·2^U4S^** or **1^I6S-Et-S4U^2** (400 nM) in the same buffers. The relative fluorescence intensity was determined after each addition. A model with one binding site and two binding equilibria was fitted to the fluorescence data from the competitive titrations using the software Scientist (Micromath), holding the known *K*_D_ (20 nM) of the 2AP-containing 36mer and M.TaqI constant.

**Re-opening of the cross-linked target base pair during the binding experiment:** A binding experiment with the cross-linked duplex **1^I6S-Et-S4U^2** in M.TaqI binding buffer without DTT was carried out as described above up to a M.TaqI concentration of 280 nM. Then, the cross-link was re-opened by adding a concentrated DTT solution (0.5 M) to reach a final DTT concentration of 1 mM in both solutions. The 2AP fluorescence decreased upon addition of DTT, indicating that re-opening of the target base pair has occurred and that the re-opened non-fluorescent duplex binds tighter. The titration with M.TaqI was then continued. The fitted binding curve from the titration of the cross-linked duplex **1^I6S-Et-S4U^2** was overlaid onto the data points before the addition of DTT, and the binding curve from the titration of duplex **1^I6S^·2^U4S^** was overlaid onto the data points after adding DTT.

## Supporting Information

File 1 Derivation of equation (1) and Figures S1 and S2.
