# Supplementary material for: Reversibly locked thionucleobase pairs in DNA to study base flipping enzymes
Source: Beilstein J Org Chem. 2014 Oct 1;10:2293–306. doi: 10.3762/bjoc.10.239 (PMC4187101; doi:10.3762/bjoc.10.239)
Supplement: File 1 — Derivation of equation (1) and Figures S1 and S2. [file Beilstein_J_Org_Chem-10-2293-s001.pdf]

Supporting Information

for

Reversibly locked thionucleobase pairs in DNA to study base flipping enzymes

Christine Beuck[1] and Elmar Weinhold[*2]

Address: [1]The Scripps Research Institute, 10550 North Torrey Pines Road, La Jolla, CA 92037, USA and [2]Institute of Organic Chemistry, RWTH Aachen University, Landoltweg 1, D-52056 Aachen, Germany

Email: Elmar Weinhold - elmar.weinhold@oc.rwth-aachen.de

*Corresponding author

Derivation of equation (1) and Figures S1 and S2

Derivation of equation (1)

The thermodynamic constants $K_D^{unlocked}$, $K_{D,init}$ and K_{flip} for the two-step binding model shown in Figure 5a are defined as follows:

$$K_D^{unlocked} = \frac{[DNA]\cdot[E]}{[E.DNA]} = \frac{[DNA]\cdot[E]}{[A]+[B]} \quad (4)$$

$$K_{D,init} = \frac{[DNA]\cdot[E]}{[A]} \quad (5)$$

$$K_{flip} = \frac{[B]}{[A]} \quad (6)$$

with [DNA], concentration of unbound DNA; [E], concentration unbound enzyme; [E . DNA] concentration of all enzyme-DNA complexes; [A], concentration of initial complex with innerhelical target base; [B], concentration of complex with flipped target base and [E . DNA] = [A] + [B].

Taking the inverse of eq. (4) and separating the terms:

$$\frac{1}{K_D^{unlocked}} = \frac{[A]+[B]}{[DNA]\cdot[E]} = \frac{[A]}{[DNA]\cdot[E]} + \frac{[B]}{[DNA]\cdot[E]} \quad (7)$$

Substituting with eqs. (5) and (6), $\frac{[A]}{[DNA]\cdot[E]} = \frac{1}{K_{D,init}}$ and $\frac{[B]}{[DNA]\cdot[E]} = \frac{K_{flip}}{K_{D,init}}$, gives

$$\frac{1}{K_D^{unlocked}} == \frac{1}{K_{D,init}} + \frac{K_{flip}}{K_{D,init}} = \frac{1+K_{flip}}{K_{D,init}} \quad (8)$$

Taking the inverse again yields equation (1):

$$K_D^{unlocked} = \frac{K_{D,init}}{1+K_{flip}} \quad (1)$$

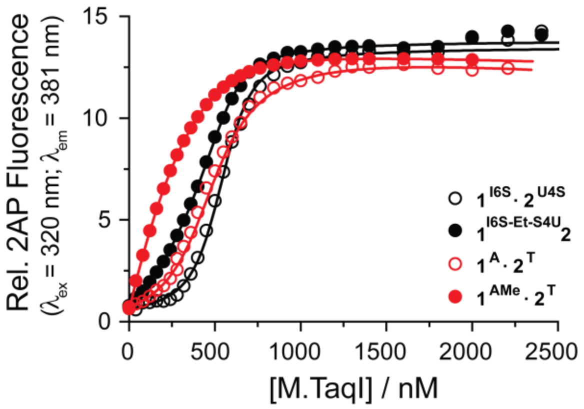

Figure S1: Direct comparison of competition binding titrations of DNA with unlocked duplex $\mathbf{1}^{\mathbf{I6S}}\cdot\mathbf{2}^{\mathbf{U4S}}$ (black open circles), locked duplex $\mathbf{1}^{\mathbf{I6S\text{-}Et\text{-}S4U}}\mathbf{2}$ (black closed cicles), A/T substrate duplex $\mathbf{1}^{\mathbf{A}}\cdot\mathbf{2}^{\mathbf{T}}$ (red open circles) and A^{Me}/T product duplex $\mathbf{1}^{\mathbf{AMe}}\cdot\mathbf{2}^{\mathbf{T}}$ (red closed circles) with M.TaqI. Increasing amounts of M.TaqI were added to a mixture of the DNA of interest and a fluorescent DNA with known K_D that contains the target base analog 2-aminopurine (2AP). A more delayed increase in the 2AP fluorescence indicates tighter binding of the non-fluorescent DNA.

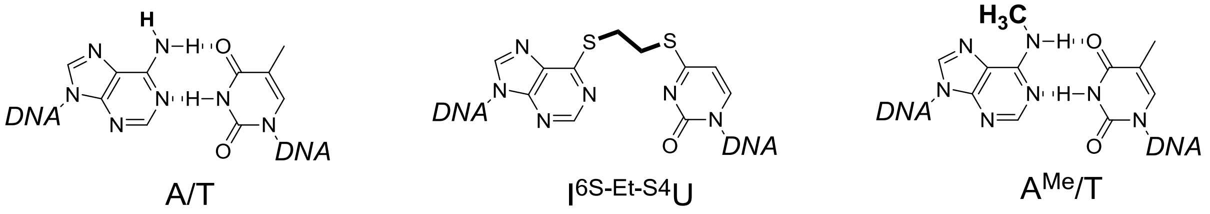

Figure S2: Comparison of the substrate A/T (left) and product A^{Me}/T (right) base pair of adenine-specific DNA MTases, with the cross-linked $I^{6S\text{-}Et\text{-}S4}U$ thionucleoside base pair (middle) used to mimic the innerhelical position of the target base in the initial M.TaqI-DNA complex before base flipping. The cross-linked base pair is missing the methyl group and therefore might be a better mimic for the A/T substrate than for the A^{Me}/T product in DNA.
